# The Effectiveness and Usability of Online, Group-Based Interventions for People With Severe Obesity: Protocol for a Systematic Review

**DOI:** 10.2196/26619

**Published:** 2021-06-30

**Authors:** Madison Milne-Ives, Dawn Swancutt, Lorna Burns, Jonathan Pinkney, Mark Tarrant, Raff Calitri, Arunangsu Chatterjee, Edward Meinert

**Affiliations:** 1 Centre for Health Technology University of Plymouth Plymouth United Kingdom; 2 Peninsula Medical School Faculty of Health University of Plymouth Plymouth United Kingdom; 3 Peninsula Dental School Faculty of Health University of Plymouth Plymouth United Kingdom; 4 Exeter Medical School College of Medicine and Health University of Exeter Exeter United Kingdom; 5 Department for Primary Care and Public Health Imperial College London London United Kingdom

**Keywords:** internet-based interventions, telemedicine, group-based interventions, obesity, severe obesity, obesity management, weight loss, weight reduction programs, diet therapy, exercise, systematic review, weight management

## Abstract

**Background:**

Globally, obesity is a growing crisis. Despite obesity being preventable, over a quarter of the UK adult population is currently considered clinically obese (typically body mass index ≥35 kg/m^2^). Access to treatment for people with severe obesity is limited by long wait times and local availability. Online and group-based interventions provide means of increasing the accessibility of obesity prevention and treatment services. However, there has been no prior review of the effectiveness of group-based interventions delivered online for people with severe obesity.

**Objective:**

The purpose of this systematic review protocol is to provide an evaluation of the effectiveness and usability of different types of online, group-based interventions for people with severe obesity.

**Methods:**

The Preferred Reporting Items for Systematic Reviews and Meta-Analyses Protocols (PRISMA-P) and the Population, Intervention, Comparator, Outcome, and Study (PICOS) frameworks were used to structure this review. The review will systematically search 7 databases: MEDLINE, Embase, the Cumulative Index of Nursing and Allied Health Literature, APA PsycNet, Web of Science, CENTRAL, and the ProQuest Dissertations and Theses databases. Two authors (MM-I and LB) will independently screen the titles and abstracts of identified articles, select studies for inclusion based on the eligibility criteria, and extract data into a standardized form. Any disagreements will be discussed and resolved by a third reviewer (EM) if necessary. Risk of bias will be assessed using the Cochrane Collaboration Risk of Bias 2 tool and a descriptive analysis will be used to evaluate effectiveness and usability.

**Results:**

The systematic review has not yet been started. It is expected to be completed and submitted for publication by December 2021.

**Conclusions:**

This systematic review will summarize the effectiveness and usability of online, group-based interventions for people with obesity. It will identify the types of online delivery that have the strongest support to help inform the development of more useful and engaging interventions for people with severe obesity.

**Trial Registration:**

National Institute for Health Research, PROSPERO CRD42021227101; https://www.crd.york.ac.uk/prospero/display_record.php?ID=CRD42021227101

**International Registered Report Identifier (IRRID):**

PRR1-10.2196/26619

## Introduction

### Background

Obesity is a serious and growing crisis; over a quarter of UK adults are considered obese (26% of men and 29% of women) [1]. Adults with a BMI greater than or equal to 35 kg/m^2^ are considered to have a very high health risk [2]. This is a significant concern, as obesity has been linked to several physical and mental health conditions, such as type 2 diabetes, heart disease, cancer, stroke, and depression [3,4]. The COVID-19 pandemic has only made obesity a more urgent health issue to tackle. Evidence to this point has identified a notably increased risk of severe COVID-19 symptoms, need for hospitalization and intensive care, and increased mortality for patients who are overweight and obese [5]. This has highlighted the need for sustained and effective interventions to improve physical activity and dietary behaviors to prevent a worsening impact on health outcomes [5].

The UK has a 4-tiered pathway for obesity services: tier 1 refers to universal obesity prevention services, tier 2 covers community-based lifestyle weight management services, tier 3 is specialist obesity services (provided by a multidisciplinary team), and tier 4 is surgery [6,7]. The higher tiers (3 and 4) are specifically targeted toward adults with high BMIs (≥40 kg/m^2^, or 35 kg/m^2^ with a comorbidity) [4].

In practice, access to these services is not universal. An All-Party Parliamentary Group found that more than one-third of people with obesity had not accessed any of these services, and almost 40% of those who did found the process moderately or incredibly difficult [8]. Additionally, of the 91% of Clinical CoMM-Issioning Groups (that tier 3 services fall under) that responded to a freedom of information request, less than 60% coMM-Issioned tier 3 services [8]. Likewise, in a survey conducted by Public Health England, people reported long waiting lists and a lack of local availability for tier 3 services [9]. A longitudinal cohort study found that almost three-quarters of people with severe obesity (BMI ≥35 kg/m^2^) did not access any treatment over the course of 7 years; this figure was lower, but still high (almost 60%), for people with morbid obesity (BMI ≥40 kg/m^2^) [10]. These statistics highlight a significant problem with access to and availability of obesity prevention services in the UK.

### Rationale

There are several strategies that can help address the need for greater accessibility of interventions for people with severe obesity. Group-based interventions may improve accessibility by potentially reducing the resources needed to provide interventions by supporting more patients with fewer staff hours required. This also has the potential to reduce waiting times to access interventions [4]. Group-based interventions are a common tool to promote health behavior change [11] and previous systematic reviews have found that group-based interventions are generally effective at promoting physical activity and weight loss [12,13].

Another strategy that is increasingly used to improve accessibility to a variety of health interventions is the use of digital and online platforms of delivery. Online delivery strategies have become increasingly common as COVID-19 restrictions have forced services to adapt the way they provide support [14]. A wide variety of platforms have been used to deliver services, with mixed feedback from participants [14]. A previous systematic review found evidence suggesting that web-based interventions are more effective at promoting and maintaining weight loss than minimal or no interventions, but that evidence of their effectiveness compared with in-person interventions is mixed [15]. Another systematic review found that online interventions were more effective at promoting weight loss in the short term, but not the long term, compared with offline interventions [16]. A systematic review of mobile-based interventions for people who are overweight or obese also found evidence of their effectiveness in primary and secondary health care settings [17], but evidence for their effectiveness in general is mixed [18].

This suggests that online platforms have potential to support effective weight loss interventions, but that further research is needed to determine best practice.

Combining these 2 strategies (ie, group based and web based) could further improve the accessibility of interventions, particularly given the COVID-19 restrictions on in-person interactions. However, no previous systematic reviews were identified that examined online, group-based interventions for people with obesity. One systematic review did review online social network weight management interventions, but only included 5 studies and was focused specifically on social networks without including other types of online interventions [19]. Several searches of keywords (“online OR digital,” “group-based interventions OR group interventions OR group behaviour change,” and “weight loss OR obesity”) on PROSPERO failed to identify any systematic reviews that are currently exploring the effectiveness of different types of online, group-based interventions for people with obesity.

Therefore, there is a need for a systematic review of studies that evaluate online, group-based interventions for people with severe obesity to determine their effectiveness, usability, and the conditions which make them most effective and engaging for participants. An overview of the different types of online, group-based weight loss interventions will make it easier to identify best practices and contribute to the development of new online, group-based interventions that can best promote and maintain weight loss.

To focus the evaluation, this systematic review will examine 2 main research questions. First, what means of delivering online, group-based behavior change interventions for adults with severe obesity is the most effective at establishing and maintaining positive health behavior changes and weight loss? Second, what are the perceptions of the acceptability, usability, and overall user experience for different online, group-based behavior change interventions for adults with severe obesity?

## Methods

### Overview

The Population, Intervention, Comparator, Outcome, and Study (PICOS) template and the Preferred Reporting Items for Systematic Reviews and Meta-Analyses Protocols (PRISMA-P) [20] will be used to structure the review and to identify appropriate Medical Subject Headings (MeSH) for the literature search (Multimedia Appendix 1). This systematic review will be composed of a literature search, article selection, data extraction, quality appraisal, data analysis, and data synthesis. This review was prospectively registered on PROSPERO (reference number: CRD42021227101).

### Eligibility Criteria

The PICOS-type framework (Table 1) is based on the research question stated above.

**Table 1:** PICOS framework.

Framework component	Description
Population	Adults (≥18 years) with severe obesity (defined for this review as BMI ≥35 kg/m^2^)
Intervention	Online, group-based interventions aiming to change health behavior relating to obesity (physical activity and dietary behavior)
Comparator	Other types of group-based interventions, including comparisons with face-to-face, phone, and other online platforms than the main intervention
Outcomes	The primary objective is to identify the types of online platforms used for group-based interventions for people with obesity and their effectiveness. Therefore, the primary outcomes will be the effectiveness of the interventions at supporting behavior changes (physical activity and dietary behavior) and weight loss. Secondary outcomes will include levels of engagement with the intervention, and patient-reported experience (including measures of acceptability, usability, or satisfaction). Other secondary outcomes—including details about the intervention design, aim, and format—will also be examined.
Study types	Studies that evaluate at least one online, group-based intervention for people with severe obesity will be eligible (including randomized controlled trials, quantitative, qualitative, cohort, and case studies). Reviews, protocols, and papers that describe interventions without evaluating them will be excluded.

### Search Strategy

Seven databases will be searched to find articles for this review: MEDLINE, Embase, CINAHL, APA PsycNet, Web of Science, the CENTRAL, and the ProQuest Dissertations and Theses databases. Key terms relating to online, group-based interventions for people with severe obesity were extracted from an initial review of the literature and used to develop the search terms and search strategy. Search terms will include MeSH terms and title / abstract keywords relating to online interventions, group-based interventions, and severe obesity. The search terms that will be used in this review are grouped into those 3 themes (Table 2) and the search string will be created using the following structure: online (MeSH OR Keywords) AND group-based (MeSH OR Keywords) AND severe obesity (MeSH OR Keywords). No date limit will be set. See Multimedia Appendix 2 for a sample search string.

**Table 2:** Search terms.

Component	MeSH	Keywords (in title or abstract)
Online	Internet-based intervention/ OR internet/ OR telemedicine/ OR videoconferencing/	Internet OR web OR online OR remote OR digital OR video* OR virtual OR technolog*
Group-based	Psychotherapy, Group/ OR Peer Group/ OR Group Processes/	(group* adj3 (based OR treatment* OR therap* OR virtual OR session* OR peer* OR support*)) OR “group intervention”
Severe obesity	exp obesity/ OR obesity management/	obesity OR obese OR specialist weight management OR Tier 3 weight management OR (BMI adj1 ("35" OR “40” OR “45”))

### Inclusion Criteria

The review will include studies that evaluate online, group-based interventions for people with severe obesity (defined for this review as BMI ≥35 kg/m^2^). Online, group-based interventions will be defined as an intervention delivered primarily online to a set of 3 or more people. Interventions that are primarily group based will be included, even if they have a small individual component. Interventions will need to target weight management for people with obesity but can focus on behavioral (eg, diet, physical activity) or physical (eg, weight loss or maintenance of weight loss) components. Interventions with comparisons to control groups with no intervention, waiting list or irrelevant interventions, minimal interventions, usual care, in-person interventions, or other online interventions will all be included.

### Exclusion Criteria

Studies of children and adolescents (participants aged < 18) will be excluded. Studies that address diet or physical activity behavior changes with a primary purpose other than managing obesity or weight loss (eg, rehabilitation after surgery, chronic obstructive pulmonary disease, diabetes) will also be excluded. Interventions that are primarily one-on-one but have a small group-based component will be excluded.

### Screening and Article Selection

The references identified from the database searches will be exported into a citation management software (EndNote X9; Clarivate Analytics) for storage and duplicate removal. There will be 2 stages of screening: (1) 2 independent reviewers (MM-I and LB) will screen the titles and abstracts and (2) 2 independent reviewers will screen the full text of the studies to determine final eligibility for inclusion. All disagreements between the reviewers will be discussed and a third reviewer (EM) will be consulted if consensus cannot be reached. A PRISMA flow diagram will be used to record the details of the screening and selection process to ensure study reproducibility.

### Data Extraction

Two independent reviewers (MM-I and another reviewer) will examine the full text of all of the included articles to extract outcomes into a predetermined form (Textbox 1). Any disagreements between the reviewers will be discussed and resolved by a third reviewer (EM) if consensus cannot be reached.

**Article information and data extraction.**

**General study information**

- Year of publication
- Country of study
- Sample demographics (including age, gender, target population)
- Initial sample size
- Analyzed sample size

**Intervention**

- Online platform
- Aim of intervention (eg, increase physical activity, improve dietary behavior)
- Group size
- Number and length of intervention sessions
- Intervention duration and follow-up periods
- Theory the intervention is based on (if any)
- Behavior change techniques [21] used in the intervention (if any)

**Evaluation**

- Outcomes measured
- Effect of intervention on behavior change outcomes
- Effect of intervention on health outcomes (eg, weight, BMI)
- Participant engagement (eg, drop-out rates, number of sessions attended)
- Acceptability
- Usability
- Participant satisfaction/feedback
- Other key performance indicators reported

### Quality Appraisal and Risk of Bias Assessment

Two reviewers (MM-I and another reviewer) will independently assess the risk of bias of all of the included studies. Any disagreements will be discussed or resolved by a third reviewer (EM). The risk of bias of the randomized controlled trials will be assessed using the Cochrane Collaboration Risk of Bias 2 tool to assign high or low risk of bias, or some concerns [22,23]. Nonrandomized studies will be assessed using the ROBINS-I tool [24]. Figures will be created to summarize the risk of bias in each study and the extent of each bias across all studies.

### Data Analysis and Synthesis

Because of the expected variety of study aims, measures, and reported outcomes, it is not likely that a meta-analysis will be feasible. However, the feasibility of a meta-analysis will be considered when studies have been assessed. A descriptive analysis will be conducted to summarize the extracted data. General study information will be summarized in a table. Outcomes relating to the intervention will be synthesized quantitatively (eg, by providing the percentage of studies that used a particular platform, theory, or behavior change technique and had a particular aim and by providing the mean, median, and range for outcomes such as group size, number of sessions, and session duration).

The primary outcomes—the effect of the intervention on behavior change and health outcomes—will be quantified by providing the percentage of studies that found significant evidence of effectiveness. Substantial evidence of effectiveness will be defined as a significantly better performance of the intervention than the comparator. Behavior change and physical health outcomes will be considered separately.

Analysis of other outcomes, such as acceptability, usability, and patient feedback will be determined upon review of the studies, as they could be analyzed qualitatively or quantitatively depending on what is reported. Any qualitative data reported by the study will be assessed using a thematic analysis to identify similarities and differences in participant responses to the interventions. The risk of bias in the studies will be considered in the synthesis.

## Results

The full systematic review is expected to be completed and submitted for publication by December 2021.

## Discussion

A systematic review of the literature on online, group-based behavior change interventions for adults with severe obesity will contribute to the establishment of guidelines for best practice. With the strained capacity to provide specialist weight management services (also known as tier 3 services), and the additional constraints of the COVID-19 pandemic on group-based and face-to-face services, a better understanding of how group-based interventions can be effectively delivered remotely will help inform and improve the development of online interventions for adults with severe obesity in the UK. Based on the data, this section will explore what conclusions can be drawn, the limitations of the systematic review, and key topics for future research.

## Appendix

Multimedia Appendix 1 PRISMA-P checklist.

Multimedia Appendix 2 Sample search.

## Abbreviations

MeSH: Medical Subject Headings
PICOS: Population, Intervention, Comparator, Outcome, and Study
PRISMA-P: Preferred Reporting Items for Systematic Reviews and Meta-Analyses Protocols
