# Supplementary material for: The Effectiveness and Usability of Online, Group-Based Interventions for People With Severe Obesity: Protocol for a Systematic Review
Source: JMIR Res Protoc. 2021 Jun 30;10(6):e26619. doi: 10.2196/26619 (PMC8280827; doi:10.2196/26619)
Supplement: Multimedia Appendix 2 [file resprot_v10i6e26619_app2.docx]

Appendix 2: Sample search strategy

| Ovid MEDLINE(R) and Epub Ahead of Print, In-Process, In-Data-Review & Other Non-Indexed Citations, Daily and Versions(R) <1946 to March 09, 2021> | | |
| --- | --- | --- |
|  |  |  |
| 1 | internet-based intervention/ | 451 |
| 2 | internet/ | 75106 |
| 3 | telemedicine/ | 26754 |
| 4 | videoconferencing/ | 1811 |
| 5 | internet.ab,kw,ti. | 56363 |
| 6 | web.ab,kw,ti. | 126058 |
| 7 | online.ab,kw,ti. | 132445 |
| 8 | remote.ab,kw,ti. | 74113 |
| 9 | digital.ab,kw,ti. | 133277 |
| 10 | video*.ab,kw,ti. | 134857 |
| 11 | virtual.ab,kw,ti. | 64852 |
| 12 | technolog*.ab,kw,ti. | 506653 |
| 13 | 1 or 2 or 3 or 4 or 5 or 6 or 7 or 8 or 9 or 10 or 11 or 12 | 1139487 |
| 14 | (group* adj3 (based or treatment* or therap* or virtual or session* or peer* or support*)).ab,kw,ti. | 268547 |
| 15 | group intervention.ab,kw,ti. | 2862 |
| 16 | group*.ti. | 216456 |
| 17 | Psychotherapy, Group/ or Peer Group/ or Group Processes/ | 48285 |
| 18 | 14 or 15 or 16 or 17 | 497535 |
| 19 | exp obesity/ | 220952 |
| 20 | obesity management/ | 136 |
| 21 | (obesity or obese).ab,kw,ti. | 319850 |
| 22 | specialist weight management.ab,kw,ti. | 28 |
| 23 | Tier 3 weight management.ab,kw,ti. | 9 |
| 24 | (BMI adj1 "35").ab,kw,ti. | 1738 |
| 25 | (BMI adj1 "40").ab,kw,ti. | 1478 |
| 26 | (BMI adj1 "45").ab,kw,ti. | 168 |
| 27 | 19 or 20 or 21 or 22 or 23 or 24 or 25 or 26 | 369891 |
| 28 | 13 and 18 and 27 | 494 |
